# Survival of teeth with external cervical resorption after Internal and External Repair: A Systematic Review 

**DOI:** 10.4317/jced.62050

**Published:** 2024-12-01

**Authors:** Juliana Sousa, Ana Beatriz Azevêdo, Rebeka Santos, Michelle Silva, Zilda Farias, Ana Paula Sobral

**Affiliations:** 1 MSc. Department of Oral and Maxillofacial Pathology, School of Dentistry. University of Pernambuco (UPE), Recife, Brazil; 2 PhD student, Department of Oral and Maxillofacial Pathology, School of Dentistry, University of Pernambuco (UPE), Recife, Brazil; 3 Associate Professor, Department of Oral and Maxillofacial Pathology, School of Dentistry, University of Pernambuco (UPE), Recife, Brazil

## Abstract

**Background:**

To analyze the survival rate of teeth affected by invasive cervical resorption after internal and external repair.

**Material and Methods:**

A search was conducted in PubMed/Medline, Web of Science, Embase, Scopus, the Cochrane Library, and gray literature at the DANS Easy Archive until September 2023. The selected studies were subjected to risk assessment of bias, and the quality of evidence was assessed using the Newcastle Ottawa Scale. The GRADE was used to analyze the certainty of evidence.

**Results:**

Three articles were included in this study. The Heithersay classification was used in all included studies. Only one study has reported on the Patel classification. Different results associated with the survival of treated invasive cervical resorption elements have been reported. Two studies reported a higher survival rate in externally repaired teeth than in internally repaired teeth. Only one study reported greater survival of theeth with external cervical resorption rate in the treatment with internal repair. The studies showed strong evidence and the certainty of the evidence was classified as very low.

**Conclusions:**

Failure rates were low in dental treatments with invasive cervical resorption for both repairs, with external repair being more promising and showing the highest survival rate in the follow-up period of at least one year.

** Key words:**External cervical resorption, external repair, interal repair, survival rate, dental treatments.

## Introduction

Root resorption corresponds to the loss of hard tooth tissue as a result of dental action (1) and is subdivided into physiological and pathological causes (2). Depending on its location, pathological resorption can be classified as external radicular or internal radicular (3). According to Andreasen’s classification, external resorption can be categorized as inflammatory by surface substitution or resorption (2,4).

External cervical resorption (ECR), also known as Invasive Cervical Resorption (ICR), is a specific class of external resorption that begins in the cervical region of the tooth (1) and is caused by the overproliferation of periodontal ligament tissues (5). ICR has a wide range of characteristics depending on factors such as location and degree of progression, and occurs more frequently in the incisors, canines, and upper and lower molars. Initially asymptomatic, it is discovered on routine radiography. However, it may also be associated with inflammation and gum bleeding (6).

Heithersay (2004) (7) developed a clinical classification of ICR for research and diagnosis, considering the degree of tissue destruction and anatomical location. This classification is two-dimensional and is evaluated using periapical radiographs, considering the extent of injury and proximity to the root canal (7,8). To provide a more accurate assessment of ICR, Patel *et al*. (2018) (6) proposed a three-dimensional (3D) classification using cone-beam computed tomography that considers the height of the lesion (coronary-apical extension), proximity to the root canal, and circumferential extension.

The severity and location of the resorption defect, as well as the ability of the tooth to be restored, influence the ICR treatment (9-12). External and internal repair of the defect with or without endodontic treatment, intentional replantation (tooth extraction and replacement of the treated dental element), periodic examination (intracTable teeth), and extraction are some of the treatment options mentioned in the literature (11,12).

In this regard, in view of the variability of treatments cited in the literature and aiming to deepen the knowledge regarding the topic, this systematic review aimed to analyze the survival rate of teeth with ICR after treatment with internal and external repair. The hypothesis of this study was that the survival of teeth with external cervical resorption after treatment with internal repair is higher than that after external repair.

## Material and Methods

-Registry protocol 

This systematic review was performed according to the guidelines of the Preferred Reporting Items for Systematic Reviews and Meta-Analyses (PRISMA) checklist (13,14) and was registered in PROSPERO under the number CRD42023455456.

-Eligibility criteria

The research questions of this systematic review were as follows: “Is the survival of teeth with external cervical resorption treated with internal repair higher than that of teeth treated with external repair?” The PECO strategy was used to guide the selection of the articles as follows: (1) Population - teeth with ICR; (2) Exposure/Intervention - internal repair of ICR; (3) Control/Comparator - external repairs of ICR; (4) Outcome/Outcome -Survival of teeth with external cervical resorption rate after treatment.

To qualify for eligibility in this review, the studies needed to address the treatment of ICR by external or internal repair and to relate to the time of survival of teeth with external cervical resorption. Studies with deciduous teeth, reviews, reports, or a series of cases were excluded.

-Search Methods 

Two independent reviewers (J.S.S.SS. and A.B.F.A.) conducted electronic searches of PubMed/Medline, Web of Science, Embase, Scopus, and the Cochrane Library until September 2023. In addition, for accessing the grey literature, the DANS Easy Archive was searched, as well as the list of references of the studies included in specific international endodontic journals, such as “International Endodontic Journal,” “Journal of Endodontics,” and “Australian Endodontic Journal” was manually searched. No date or language restrictions were applied in the search strategy ( Table 1).

EndNote Online (https://access.clarivate.com/login? app=endnote) was used to remove duplicates and select the included studies. Two reviewers considered the exclusion of duplicates and read the titles and summaries of the articles. If the title or summary contained insufficient information, the full article was read. Studies were excluded if they did not meet the inclusion criteria or were not fully available. All differences in the selection process between the researchers were resolved by a third reviewer (Z.B.B.M.F.) to reach a consensus through discussion.

-Data collection process

One reviewer collected data from the included studies, and a second reviewer analyzed all the extracted data. A third reviewer examined all differences in choices between the authors, reaching a consensus. The variables collected were authorship, year of publication, country of origin, study design, number of participants, sex, age, number of teeth with ICR treated by internal or external repair, classification of ICR, number of endodontically treated teeth, type of repair, follow-up period, and rate of survival of teeth with external cervical resorption.

-Evaluation of the quality of included studies and evidence

To analyze the methodological quality of the included articles, the Newcastle-Ottawa Scale(15) tool, which contains eight items for evaluating the methodological quality of retrospective observational studies, was used. This tool considers three perspectives: participant selection, comparability between groups, and the outcome of interest. The Grading of Recommendations Assessment, Development, and Evaluation (GRADE) approach was used to analyze the certainty of evidence. Assessment according to the GRADE approach was based on the risk of bias, inconsistency, indirect evidence, inaccuracies, and publication bias. Thus, the certainty rate of the evidence was categorized as high, moderate, low, or very low (16).

## Results

-Result of the Search Process 

**Figure 1:**
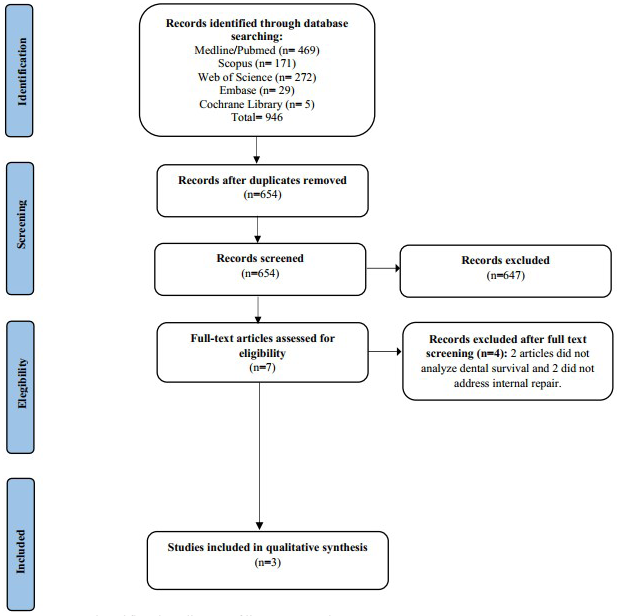
PRISMA-based flowchart diagram of literature Search.

After searching the databases, 946 articles were identified and allocated as follows: PubMed (n = 469), Scopus (n = 171), Web of Science (n = 272), Embase (n = 29), and the Cochrane Library (n = 5). Searches were also conducted in the Dans Easy Archive, Journal of Endodontics, International Endodontics Journal, and Australian Endodontics Journal. After the duplicates (n = 292) were removed, 654 articles remained for reading titles and summaries. After applying the eligibility criteria, only three articles remained to be read in full, and all were included in the systematic review (Fig. 1).

-Methodological Characteristics of the Included Studies 

The general characteristics of the items included are summarized in  Table 2.  Table 3 shows the relationship between the type of repair and survival of teeth with external cervical resorption.  Table 4 highlights the risk of bias according to the Newcastle-Ottawa Scale (15), and the level of evidence is shown in  Table 5 according to the GRADE assessment (16).

-Profile of the sample studied and classification of the affected teeth with ICR

In relation to the sample profile, a male predominance of patients affected by ICR was observed, except in a study by DeLuca *et al*. (2023) (19), which reported a higher incidence in females. The ages of the participants ranged from 12 to 89 years. With regard to the classification of ICR, Heithersay’s classification was present in all included studies, and in the study by Irinakis *et al*. (2022) (7,17), there was a higher frequency of Class II (a well-defined invasively resorbing lesion that penetrated near the coronal pulp chamber but showed little or no extension in the root dentin). Mavridou *et al*. (2022) (18) showed a higher frequency of class III (deeper dentin invasion by resorbing tissue, not only involving the coronal dentin, but also extending at least to the coronal third of the root), and DeLuca *et al*. (2023) (19) showed a greater predominance of class IV (large invasive resorption, a process that extends beyond the coronal third of the root canal).

Only DeLuca *et al*. (2023) (19) reported the Patel classification, with 3Dp (resorption height extending up to the middle third of the root, circumferential extension 270, and likely pulp involvement) being the most prevalent classification (n = 23; 16.20%), followed by 3Bp (resorption heights extending to the mid-third of the roots, peripheral extension 90, and probable pulp inclusion) (n = 19; 13.38%).

-Survival rate of teeth treated with internal and external repair

Different results associated with the survival of elements treated with ICR were observed in the articles included in this review ( Table 3). Studies by Mavridou *et al*. (2022) (18) and DeLuca *et al*. (2023) (19) reported a higher survival of teeth with external cervical resorption rate in externally repaired teeth than in internally repaired teeth; the first study reported a survival of teeth with external cervical resorption rate of 50% in externally repaired teeth, whereas the survival rate of internally repaired teeth was 20%. DeLuca *et al*. (2023) (19), despite not citing the total survival rate of teeth treated with internal and/or external repair (n = 142), reported an isolated sample of teeth that survived for the longest period in their study: eight dental elements survived external repair, and five survived internal repair. The study by Irinakis *et al*. (2022) (17) was the only one that showed higher survival of teeth with external cervical resorption in treatment with internal repair, with a rate of 78.3%, whereas the index for external repair corresponded to 62.5%. Of the three studies (17-19), only Irinakis *et al*. (2022) (17) reported the number of endodontically treated teeth.

-Assessment of the quality of studies and evidence

The studies were evaluated according to the Newcastle-Ottawa Scale (15) ( Table 4). Overall, they provided strong evidence, producing positive answers to six of the nine questions. For item 2 of the dimensions “selection” and “comparability,” there was no score because the articles presented no control for comparison.

Additional information ( Table 5) demonstrate the GRADE assessment (16) related to the outcome of the survival rate of teeth affected by ICR treated through internal or external repair. All three studies (17-19) were included, and the certainty of the evidence was rated as very low. The areas in which reduction was observed were ‘inconsistency,’ ‘indirect evidence,’ and ‘inaccuracies’.

Discussion 

Based on the analyzed results, a higher survival of teeth with external cervical resorption rate - affected teeth was observed in externally repaired teeth than in internally repaired teeth. Thus, the hypothesis that the survival of teeth with external cervical resorption rate after internal repair is higher than that after external repair was not accepted, with the null hypothesis prevailing.

The articles included in this review addressed the treatment of ICR using external and internal repair and were related to survival of teeth with external cervical resorption time. Despite the small number of articles and participants included in the final sample, this study is promising and unprecedented. Currently, the treatment prognosis is supported by limited scientific evidence (17). Thus, it was possible to analyze, through a systematization of data, without considering the local determinants, that failure rates were low in dental treatments with ICR for both repairs, with external treatment being the most promising, and showing the highest survival rate in the follow-up period. This review provides new perspectives and potential collaborations regarding universally accepted ICR treatment strategies (17-19).

Among the included studies, only the study by DeLuca *et al*. (2023) (19) showed a greater predilection for female sex in patients affected by ICR. There was a predominance of males in other studies(17,18). These findings corroborate those of other studies, such as Jeng *et al*. (2023) (20), who investigated the prognosis and potential factors of teeth affected by ECR after surgical intervention for external repair, with or without endodontic treatment. They identified that 26 of 42 affected patients were male. Their findings also showed that sex and other variables, such as age, dental position, and need for endodontic treatment, did not have a statistically significant effect on the prognosis of the evaluated sample. Another study by Mavridou *et al*. (2017) (21), whose aim was to analyze the occurrence of ECR in relation to patient characteristics, identified 337 affected teeth, of which 54% were found in male patients. They noted that ICR data were not related to the patient’s sex, but rather to its incidence and predisposing factors. This suggests that age does not influence disease onset.

Regarding the age of the affected patients, there was a trend for those in their fourth decade of life. This was also observed by Jeng *et al*. (2023) (20). This is because of the slow evolution of resorption. They are asymptomatic and are identified during routine examinations (11). This leads to a delay in diagnosis, which is usually performed when patients reach the fourth decade of life. The teeth most frequently affected were the anterior upper and first upper molars and lower molars (17-19), as well as other findings in the literature (8,21).

It was unanimous that the study included a minimum follow-up period of one year after treatment. Only two authors quoted the average follow-up time (17,19). In contrast, Heithersay *et al*. (1999) (22) reported a minimum follow-up time of 3 years when evaluating success. Jebril *et al*. (2020) (23) performed a minimum 20-month postoperative follow-up. Mavridou *et al*. (2020) (18) further added that long follow-up times are essential to draw certain conclusions because separation becomes more pronounced after the first 5 years. These variations in follow-up time demonstrate the need for minimum time standardization of powders and teeth carrying ICR with or without treatment. As this is a challenging topic, when an ICR injury cannot be predictably repaired, periodic review is a favorable treatment option (19).

In studies by Mavridou and DeLuca *et al*. (2023) (18,19), both repairs showed survival rates; however, it was higher for external repair. Irinakis *et al*. (2022) (17) reported higher survival of teeth with external cervical resorption rates for internal repair in contrast to the results of the aforementioned authors. Nevertheless, this difference identified by Irinakis *et al*. (2022) (17) was not significant. Moreover, the numerical differences in a few studies and the small number of samples did not allow us to fully answer the clinical questions associated with the subject. This suggests the need for new studies that seek to explain, in larger numbers, the percentages related to the survival rates of teeth with ICR after internal or external repairs to elucidate the hypotheses of the evidence and promote better conduct of the treatments.

While quoting the survival rate, success rate, and causes of failure, the included authors did not emphasize the numbers associated with internal or external repairs. As a result, some results are generalized, such as those of De Luca *et al*. (2023) (19): two cases survived for 10 years, 10 cases lived for 6–9 years, 13 cases for 3–5 years, 9 cases for 2 years, and 16 cases for 1 year. Irinakis *et al*. (2022) (17) generally stated that 24 teeth were categorized as faults and pointed to their causes. Thus, it is not possible to determine to which category or group the teeth treated with repairs belong. This may be seen as a limitation of the current study, indicating the need for further research with this focus.

As for the classifications and affected teeth, the studies included were conducted according to the classifications of Heithersay (17,18) or Heitheray and Patel (19). According to the Heithersay classification (7), the classes with the highest number of affected teeth were II (17), III(18), and IV (19). Overall, this diagnosis is most often made in more advanced stages based on the absence of pain or a complaint from the patient, which brings challenging forecasts.

Moreover, the varied classifications in each study were due to different factors, such as professional experience and calibration, local factors related to the patient or injury, entrance door, or surfaces involved. This could have influenced the outcomes of the proposed treatment. Thus, the outcome of treatment may be conditioned by factors other than classification. That is, the classification, whether assertive or not, and proposed treatment may be confused with the outcome. For example, in the study by De Luca *et al*. (2023) (19), classification was not associated with treatment results. According to Heithersay (1999) (22), the success rate decreases as an injury progresses from class I or II to class III or IV. Jebril *et al*. (2020) (23) found that cases with the highest success rates were in Heithersay classes I–III when local factors were not considered.

Mavridou *et al*. (2022) (18), before planning the treatment, took several criteria into account, including the patient’s age, potential etiological factors, tooth type, pain sensation, probability of probe, location and size of the entry portals, aesthetics, pulp and periodontitis, status, occlusion, and joint. However, the classification was based on that of Heithersay *et al*. (2004) (7), as Patel *et al*. (2018) (6) did not exist at that time. It is important that for new studies on the subject, a classification of lesions based on a 3D classification, Patel, is used to prove its reliability and provide additional information about the extent of the injury, which can influence treatment planning.

The location and extent (class) of resorption and the location and size of the entrance door are important factors in deciding the treatment approach, whether external, internal, or non-intervention. Smaller resorptions and where the dental pulp is still vital are best treated by external repairs, which increase the survival of the affected teeth, while internal repairs are better indicated for teeth with more extensive resorptions. Only DeLucca *et al*. (2023) (19) used Patel *et al*. (2018) (6) classification. This is considered a more assertive diagnosis because it characterizes a 3D extension. For this author, some teeth that survived the longest in this study were those that did not receive treatment. According to Irinakis *et al*. (2022) (17), the lowest failure rates are related to teeth with ICR Heithersay classes I and II (7). The highest failure rate was observed in class IV, where the probability increased substantially after the fourth year. Thus, the better the classification of resorption and the lower the need for intervention, the better is the prognosis.

Regarding methodological quality, according to the Newcastle-Ottawa scale (15), the included studies demonstrated strong evidence. Nevertheless, there were items for which the negative answers needed to be discussed. Item 2 of the selection and comparability items presented negative responses owing to the absence of comparative control groups. This fact denotes an important limitation of this review, which involved a small number of articles in the final sample of articles, none of which were presented as case-control studies due to the lack of other study drawings available in the literature. The small number of studies involved observational drawings, which contributed to a very low certainty of evidence as evaluated using the GRADE system (16). In addition, the small number of participants in the sample corroborated the inaccuracies of these studies.

This systematic review was conducted using internationally recognized databases, and it was possible to broaden the knowledge on the subject through the searches and selections made. Therefore, this review presented as a construction of considerable impact because it achieved the objectives envisaged. Our results can assist professionals in the classification, diagnosis, treatment, and prognosis of ICR treated with external and internal repair, consequently increasing the survival rates of affected dental elements. Thus, more research with larger samples and other study designs should be conducted to deepen our understanding and guide safer treatments.

## Conclusions

Monitoring of lesions by teeth with external cervical resorption should be carried out in order to make a decision for treatment.

An external repair is generally used for teeth with minor lesions, particularly those with normal pulp, with consequent greater survival of teeth with external cervical resorption.

In contrast, an internal repair is preferred when treating advanced or more extensive lesions.

Owing to the relevance of this topic, this systematic review serves as an incentive to conduct more clinical research addressing the types of interventions, with a longer follow-up time and larger sample size.

## Figures and Tables

**Table 1:** Search strategy.

Database	Search strategy	Filter
PubMed/MEDLINE (469)	(external cervical resorption OR invasive cervical resorption OR invasive cervical root resorption OR cervical root resorption OR external cervical tooth root resorption AND therapeutic OR therapy OR treatment) #1 AND #2	No filters applied
Web of Science (272)	ALL= (external cervical resorption OR invasive cervical resorption OR invasive cervical root resorption OR cervical root resorption OR external cervical tooth root resorption) AND ALL=(therapeutic OR therapy OR treatment) #1 AND #2	No filters applied
Embase (29)	('external cervical resorption'/exp OR 'external cervical resorption' OR 'invasive cervical resorption'/exp OR 'invasive cervical resorption' OR 'invasive cervical root resorption' OR 'cervical root resorption' OR 'external cervical tooth root resorption') AND ('therapy'/exp OR therapy OR 'treatment outcome'/exp OR 'treatment outcome')	No filters applied
Scopus (171)	TITLE-ABS-KEY (external cervical resorption OR invasive cervical resorption OR invasive cervical root resorption OR cervical root resorption OR external cervical tooth root resorption) AND TITLE-ABS-KEY (therapeutic OR therapy OR treatment)	Article title, Abstract, Keywords
Cochrane Library (5)	("external cervical resorption" OR "invasive cervical resorption" OR "invasive cervical root resorption" OR "cervical root resorption" OR "external cervical tooth root resorption") AND (therapeutic OR therapy OR treatment)	Trials
	Grey Literature	
DANS Easy Archive	("external cervical resorption" OR "invasive cervical resorption" OR "invasive cervical root resorption" OR "cervical root resorption" OR "external cervical tooth root resorption") AND (therapeutic OR therapy OR treatment)	All studies
Journal	Search strategy	Filter
Journal of Endodontic	"external cervical resorption" OR "invasive cervical resorption" AND treatment	No filters applied
International Endodontic Journal	"external cervical resorption" OR "invasive cervical resorption" AND treatment	No filters applied
Australian Endodontic Journal	"external cervical resorption" OR "invasive cervical resorption" AND treatment	No filters applied

**Table 2:** Characteristics of the included studies related to sex, age, and classification of teeth affected by ICR.

Author/Year/Country	Study Design	Sample size	Sex	AR, M e SD of age	Nº of teeth with ICR treated with ER or IR	Heithersay classification	Patel classification
Irinakis et al. (2022) (17)Canada	Cohort	67	Male: 39Female: 28	FE: 12-89 M: no infoDp: no info	39	Class I: 11Class II: 35Class III: 21Class IV: 22	No info
Mavridou et al. (2022) (18)Belgium	Cohort	307	Male: 205Female: 155	No info	139	Class I: 53Class II: 98Class III: 116Class IV: 72No info: 21	No info
DeLuca et al. (2023) (19)USA	Cohort	118	Male: 53 Female: 65	FE: 15-87M: 49 Dp: 18	61	Class I: 7Class II: 16Class III: 50Class IV: 69	1Ad: 61Ap: 22Ad: 62Ap: 72Bp: 82Cd: 12Cp: 13Ad: 43Ap: 73Bd: 13Bp: 193Cp: 93Dp: 234Bp: 54Cp: 34Dp: 10No info: 30

AR: Age range; M: Mean; SD: Standard Deviation; ICR: Invasive Cervical Resorption; ER: External repair; IR: Internal repair

**Table 3:** Main survival of teeth with external cervical resorption outcomes associated with the type of treatment.

Author/Year/Country	Nº of teeth with ICR treated with IR or ER	Nº of endodontically treated teeth	Repair type	Mean follow-up	Minimum follow-up time	Maximum follow-up time	Survival of teeth with external cervical resorption
Irinakis et al. (2022) (17)Canada	39	54	Internal repair (n=23) External repair (n=16)	3,9 years	12 months	10 years	78.3% of the teeth treated through internal repair and 62.5% of the external repair survived, however, this difference was not significant.
Mavridou et al. (2022) (18)Belgium	139	No info	Internal repair (n= 47) External repair (n=92)	No info	12 months	10 years	Nearly 20% of the teeth treated with IR and 50% of the teeth treated with ER survived.
DeLuca et al. (2023) (19)USA	61	No info	Internal repair (n=26) External repair (n=35)	3,4 years	12 months	10 years	The teeth that survived for the longest period of time in the study were: 3 treated through IR and 8 treated via ER.

ICR: Invasive Cervical Resorption; ER: External repair; IR: Internal repair

**Table 4:** Risk of bias to the Newcastle Ottawa scale.

Studies	Study Design	Items								
		Selection				Comparability	Exposure			Score
		1	2	3	4	1	1	2	3	
Irinakis et al. (2022) (17)	Retrospective Cohort	☆	-	☆	☆	-	☆	☆	☆	6
Mavridou et al. (2022) (18)	Retrospective Cohort	☆	-	☆	☆	-	☆	☆	☆	6
De Luca et al. (2023) (19)	Retrospective Cohort	☆	-	☆	☆	-	☆	☆	☆	6

Strong evidence - consistent findings among several high-quality studies 6/9; moderate evidence - consistent findings between several poor quality studies and/or a high quality study 4-5/9; limited evidence - one study of lower quality< 4; contradictory evidence - inconsistent finding between multiple studies; no evidence - no evidence between studies.

**Table 5:** GRADE table of evidence of the survival rate of the teeth with invasive cervical resorption treated by internal or external repair as outcome.

Certainty assessment							Certainty
№of studies	Study Design	Risk of Bias	Inconsistency	Indirectness	Imprecision	Other Considerations	
3	Observational study	Not serious	Not serious	Not serious	Serious^a^	Publication bias strongly suspected^b^	⨁◯◯◯Very low

a All the articles included presented a small number of participants (less than 400). Only one has a number greater than 300 b All the included studies are small and of observational design (cohort) Source: https://gdt.gradepro.org/app/

## Data Availability

The datasets used and/or analyzed during the current study are available from the corresponding author.
